# Changes in older people’s quality of life in the COVID-19 era: a population-based study in Finland

**DOI:** 10.1007/s11136-022-03210-2

**Published:** 2022-09-04

**Authors:** Sini Siltanen, Katja Ilmarinen, Minna-Liisa Luoma, Suvi Leppäaho, Sari Kehusmaa

**Affiliations:** The Finnish Institute for Health and Welfare, PO Box 30, 00271 Helsinki, Finland

**Keywords:** Wellbeing, Lifestyle, Aging, Coronavirus, Resilience, Public health

## Abstract

**Purpose:**

We investigated how quality of life (QoL) changed between 2018 and 2020, and how its related factors, i.e., communication with friends and family, loneliness, and sleeping difficulties changed amid the early-phase COVID-19 pandemic among Finnish older people.

**Methods:**

This study utilizes data from a repeated cross-sectional, population-based FinSote survey in 2018 and 2020. Participants were community-dwelling people aged 75 years or older (*N* = 9781 in 2018 and *N* = 9919 in 2020). QoL was assessed with the EUROHIS-QoL-8 scale. Changes in QoL-related factors were self-evaluated in 2020. Statistical methods included *t* test, Cohen’s *D*, and chi-square test. To identify potential risk groups, all analyses were stratified by socio-demographic features including sex, age, economic deprivation, living alone, and difficulties in Instrumental Activities of Daily Living (IADL).

**Results:**

QoL improved slightly from 2018 to 2020 (means 3.68 and 3.81, respectively). Only those reporting economic deprivation demonstrated a slight decrease in QoL (3.24 vs. 3.14). Of respondents, 63% reported having less communication with friends and family, 42% having felt lonelier, and 20% having more sleeping difficulties amid the pandemic. Negative changes were more often reported by women, the oldest old, those living alone, reporting economic deprivation, or manifesting IADL difficulties.

**Conclusion:**

Finnish older people’s QoL was not affected as much as expected amid the pandemic, although some population groups were, however, more susceptible to the negative effects of the pandemic on QoL-related factors. Results imply that various socio-demographic features may shape the effects of a global pandemic and its control measures on wellbeing.

## Background

Quality of life (QoL), as defined by the WHO, represents “individuals’ perception of their position in life in the context of the culture and value systems in which they live and in relation to their goals, expectations, standards and concerns” [1]. Hence, it is a highly subjective phenomenon reflecting one’s level of satisfaction or displeasure towards the important aspects of their life [2]. QoL incorporates many life areas, such as physical health, psychological state, level of independence, social relationships, and personal beliefs [1]. In older age, especially the roles of personal health and social relationships are emphasized [3]. Other known correlates of greater QoL include having good mobility, engaging in leisure and social activities, having a positive psychological outlook [4–6], and being physically active [7].

In spring 2020, a notable threat for population’s QoL emerged as the COVID-19 coronavirus disease started to spread in Finland. When the Finnish Government declared a state of emergency in March 2020, many social distancing measures were adopted, for example public gatherings were limited to few people, unnecessary traveling was prohibited, and e.g., libraries, museums, leisure centers, and sport facilities were closed down [8]. Furthermore, as older people were found to be especially vulnerable for severe COVID-19 infections [9, 10], the government laid out a general guideline according to which all people aged over 70 years should avoid physical contact with others outside one’s household [8]. Since the outbreak more than a million COVID-19 cases (population 5.5 mil.) and about 4500 deaths have been reported in Finland [11]. The control measures have been gradually loosened over time and the age-based recommendation to adopt quarantine-like conditions has been lifted. In general, Finland has survived the pandemic well as the number of deaths has remained rather low throughout the pandemic compared to neighboring countries Sweden and Estonia, also economies fared well [12].

As expected, in the early-phase COVID-19 pandemic, older people’s physical and social activity outside their homes decreased, loneliness increased, and they started to report more sleep problems [6, 13–16]. Despite the negative changes in these QoL-related factors, quality of life itself remained rather stable [6, 17]. However, whether the pandemic has affected older people’s QoL in the later phases when the social distancing measures have been prolonged, has hitherto remained unclear. It is possible that older people, who seem to have a better capacity to cope with environmental stressors than younger people [18], have adapted to the control measures and restricted lifestyle for example by compensating decreased social activity by increasing physical activity [19]. However, it is also possible that the prolonged restrictions and psychosocial burden have gradually exposed to more severe conditions and greater negative changes in lifestyles and eventually, in QoL [20]. Moreover, it is not clear yet whether the potential change in QoL has been similar between different population groups and whether there are some demographic factors, such as age, sex, socioeconomic status, functional capacity or living situation, that make some older people particularly vulnerable. Based on the findings from studies conducted in the early-phase COVID-19 in Finland, it seems that negative changes at least in loneliness and social activity were more pronounced among the oldest old and those living alone [15].

The objectives of this study were (1) to assess to which extent overall QoL has changed between 2018 and 2020 by using repeated cross-sectional data collected in these two time periods (before COVID-19 pandemic and during the early-phase pandemic), and whether these changes differ between various population groups, and (2) investigate people’s self-evaluated changes in QoL-related factors, including keeping in touch with friends and relatives, feeling lonely, feeling optimistic about the future, daily exercise, alcohol use and sleeping difficulties.

## Methods

### Data, design, and population

This study utilized data from the FinSote National survey (www.thl.fi/finsote) conducted in 2017–2018 and 2020–2021 in the Finnish Institute for Health and Welfare. The FinSote National survey is a nationally representative study assessing health, wellbeing and service use of the Finnish population by different population groups and regions [21]. The survey is based on a random sampling design, which is stratified by age and region and drawn from the Digital and Population Data Services Agency. In the sampling phase, those persons who have been included in the sample in previous years are excluded, and therefore, the study samples do not contain the same individuals across years. Data are collected from three age groups, 20–54, 55–74, and + 75-year-olds, by postal and online questionnaires, which are available in four different languages (Finnish, Swedish, Russian, and English). Data are collected every other year. In 2017–2018, the data were collected between October 2017 and March 2018, and in 2020, between September 2020 and February 2021. Hereinafter, we use 2018 and 2020 to describe the time points accordingly to the year in which most of the responses were received.

In the present study, we report results only for participants aged 75 years or older. In 2018, totally 9781 people belonging to this age group responded to the survey, rendering a response rate of 57%. In 2020, the respective numbers were 9919 and 59%.

### Study variables

Quality of life was assessed in 2018 and 2020 with the validated EUROHIS-QOL-8 scale, which is a shortened 8-item version of the WHOQOL-BREF [22, 23]. The items forming a one-factor structure are: (1) How would you rate your quality of life? (2) How satisfied are you with your health? (3) Do you have enough energy for everyday life? (4) How satisfied are you with your ability to perform your daily living activities? (5) How satisfied are you with yourself? (6) How satisfied are you with your personal relationships? (7) Have you enough money to meet your needs? and (8) How satisfied are you with the conditions of your living place? The items are answered by using individualized five-point Likert scales (e.g., from “not at all” to “completely”) with higher scores indicating a more positive response. In the present study, we utilized the EUROHIS-QOL-8 index, which is calculated by summing all the individual item responses and then dividing the sum by the number of responded items. The index ranges between 1 and 5 with higher scores indicating greater QoL.

Quality of life-related factors included keeping in touch with friends and relatives, feeling lonely, feeling optimistic about the future, daily exercise, sleeping difficulties, and alcohol use. These items were assessed in 2020 from about 80% of the participants (while the remaining 20% received questions related to digitalization). Changes in QoL-related factors were assessed with the question “Have the coronavirus pandemic or the subsequent restrictive measures affected your everyday life?” and the response options were “No influence”, “Yes, decreased”, “Yes, increased” and “Not applicable” [21]. Since the last response option was guided to be chosen only in case if the item was not a part of the participant’s life at all, we decided to exclude the “Not applicable” responses from the analyses (see Table 3). Further, we converted the remaining response options to reflect a positive, negative, or no change.

### Variables defining different population groups

We used sex, age group, educational level, urbanization of place of residence, economic deprivation, living alone, IADL difficulty and use of Internet as factors representing different population groups. All these variables were categorized into two categories. Age, sex and living area (urbanization) were drawn from the Digital and Population Data Services Agency. Age was categorized into 75–84 vs. + 85 years old. Urbanization was initially trichotomous describing people who live (1) in the city, (2) in an urban area, or (3) in the countryside, but we subsequently combined the first two categories. Educational level, economic deprivation, living alone, IADL difficulty, and use of Internet were self-reported and collected with the postal questionnaires. Educational level was calculated based on self-reported years of full-time education and categorized initially into three: low education (approximately 0–8 years), moderate education (approx. 9–12 years) and high education (approx. 13 years or more). We then combined the moderate and high education categories. Economic deprivation was assessed with a question “Have you within the past 12 months ever…” including three items: (1) feared that you will run out of food before you can get money to buy more, (2) been unable to buy medicines because you did not have any money, and (3) not visited a doctor because you did not have any money? Response options were “yes” and “no”, and people who responded “yes” to at least one of these three items were categorized as reporting economic deprivation. Living alone was assessed with a question with two response options “yes” and “no”. Difficulties in instrumental activities of daily living (IADL) were assessed with four individual items: (1) light housework (vacuum cleaning, washing dishes, making beds, doing laundry, etc.), (2) minor repairs around the home (replacing a light bulb or a smoke alarm battery, etc.), (3) day-to-day financial transactions (paying bills, withdrawing cash, etc.) and (4) shopping for food. Response options were “yes, with no difficulty”, “yes, with some difficulty”, “yes, but with great difficulty”, “no, I cannot”. Persons who chose either of the last two response options at least to one of the four items were categorized as having IADL difficulty. Finally, Internet use was assessed with a question “Do you use Internet for the following: e-services (e.g., My Kanta, MyTax, the Social Insurance Institution of Finland [Kela])”. Response options were “I use it independently”, “I use it with another person’s help or someone else uses it on my behalf” and “I don’t use”. People who reported using Internet independently or with the help of another person were categorized as using Internet.

### Statistical methods

All analyses were conducted with the IBM SPSS Statistics version 27. In the analyses, we utilized the complex samples method and weight coefficients for the random sample to represent the whole Finnish older population. The differences in mean QoL scores between 2018 and 2020 were tested with *t* test. Effect sizes were calculated using Cohen’s *D* to communicate the practical significance of results obtained from the *t* test [24]. The Kolmogorov–Smirnov test was used to test the normality of the distribution in the QoL scores. Differences in self-evaluated changes in QoL-related factors during the COVID-19 pandemic, i.e., keeping in touch with friends and relatives, feeling lonely, feeling optimistic about the future, daily exercise, sleeping difficulties and alcohol use, were analyzed using crosstabs and tested with chi-square test. All analyses were stratified by sex, age group, educational level, urbanization, economic deprivation, living alone, IADL difficulty and Internet use.

## Results

Background characteristics of the FinSote 2020 study participants are presented in Table 1. The table contains separate columns for raw data and for data analyzed with weight coefficients and complex samples method. The mean age of participants in 2020 was 80.6 (standard deviation, SD 4.7). As the survey is nationally representative, the proportions presented in the weighted sample column resemble those found also in the FinSote 2018 data.

**Table 1:** Background characteristics of the FinSote 2020 survey participants

	Raw data (*N* = 9052–9919)		Weighted sample (*N* = 3265–3603)	
	*N*	%	*N*	%
Sex, women	5675	57	2172	60
Age group, 75–84 years	7942	80	2585	72
Education, high or moderate	5727	63	2008	62
Living in an urban area, yes	8146	82	3007	84
Economic deprivation, yes	581	6	224	6
Living alone, yes	4099	44	1681	49
Uses internet to run errands, yes	5113	55	1748	52
Self-reported memory, poor or very poor	603	6	246	7
IADL difficulty, severe or unable	2995	30	1223	35

Proportions presented in column “Weighted sample” have been analyzed by using weight coefficients and the complex samples method and can be considered as representing the whole Finnish older population

### Changes in quality of life between 2018 and 2020

As Table 2 shows, the mean QoL index score among all study participants was around 3.7 (range 1–5) in 2018 and 3.8 in 2020, indicating a slight increase between these 2 years (*p* < 0.001 obtained from *t* test). Similar increases were observed for all population groups, except for those reporting economic deprivation. Among those who reported economic deprivation, QoL was at a lower level to start with and over time, decreased a little (*p* = 0.036). Despite the statistical significance in all models, the effect sizes calculated using Cohen’s *D* ranged from 0.11 to 0.25 [23].

**Table 2:** Quality of life scores (EUROHIS-QoL-8 index, range 1–5) in 2018 and 2020 by different population groups of the FinSote 2020 survey

	2018		2020		Difference	
	Mean	SE	Mean	SE	*p* value	Effect size
All	3.68	0.009	3.81	0.008	< 0.001	0.20
Men	3.7	0.010	3.84	0.011	< 0.001	0.22
Women	3.67	0.012	3.79	0.010	< 0.001	0.19
75–84 years	3.75	0.010	3.87	0.008	< 0.001	0.22
85 years or over	3.54	0.020	3.65	0.018	< 0.001	0.13
High or moderate education	3.74	0.013	3.88	0.010	< 0.001	0.22
Low education	3.63	0.014	3.73	0.013	< 0.001	0.14
Living in urban area	3.69	0.010	3.82	0.008	< 0.001	0.21
Living in rural-like area	3.67	0.016	3.78	0.017	< 0.001	0.17
No economic deprivation	3.73	0.009	3.86	0.008	< 0.001	0.21
Economic deprivation	3.24	0.033	3.14	0.035	0.036	0.11
Living with someone	3.73	0.012	3.88	0.010	< 0.001	0.25
Living alone	3.64	0.014	3.75	0.012	< 0.001	0.16
No or moderate IADL difficulty	3.88	0.009	4.00	0.008	< 0.001	0.23
Severe IADL difficulty or unable	3.32	0.017	3.46	0.015	< 0.001	0.20
Uses internet to run errands	3.83	0.014	3.92	0.010	< 0.001	0.16
Does not use internet to run errands	3.59	0.012	3.70	0.012	< 0.001	0.16

*p* value for difference in estimated means between 2018 and 2020 in each population group category, tested with *t* test. Effect size calculated using Cohen’s *D*

*IADL* instrumental activities of daily living

### Self-evaluated changes in keeping in touch with friends and relatives, feeling lonely, and feeling optimistic about the future

**Fig. 1:**
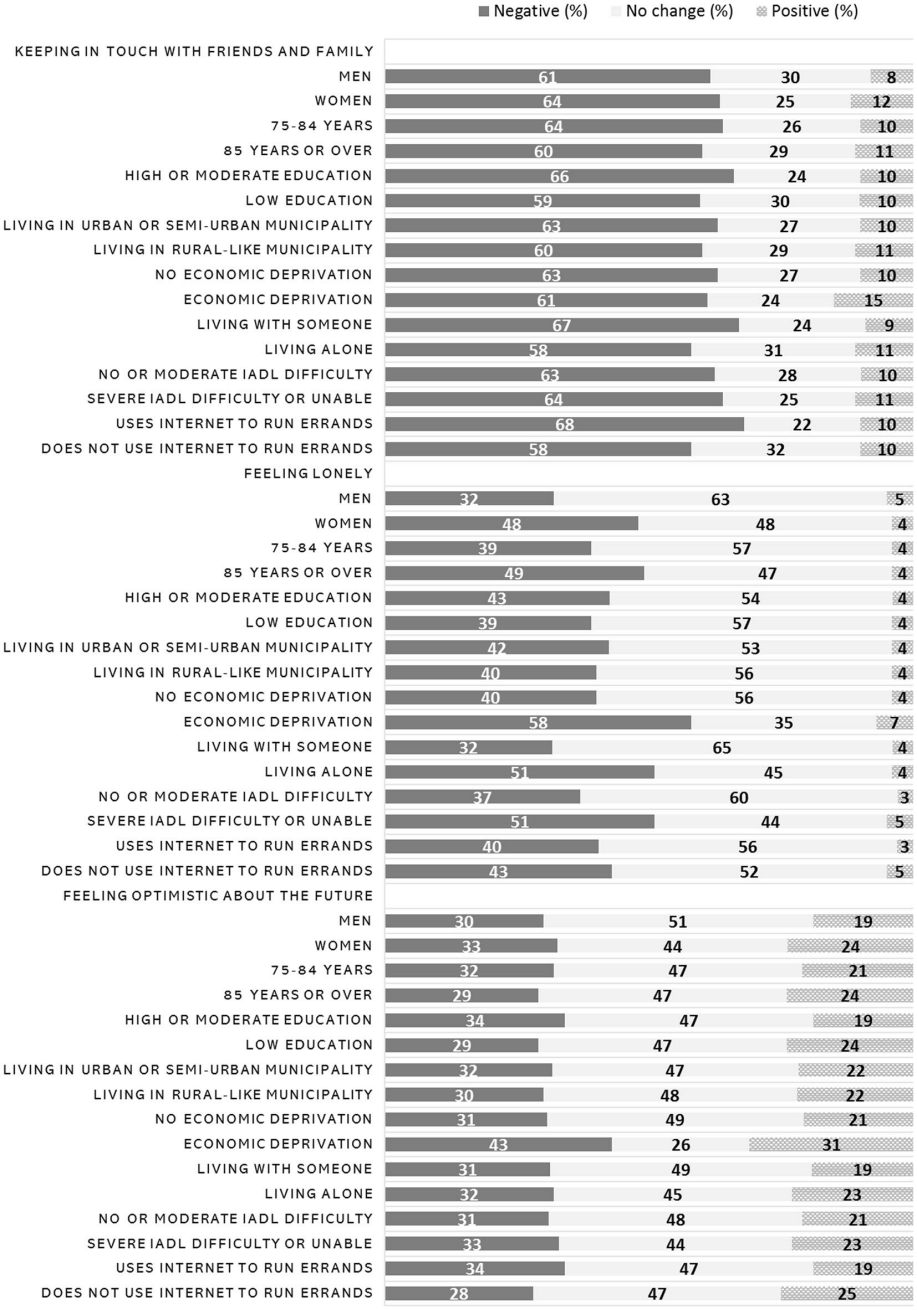
Older people’s self-evaluated changes in keeping in touch with friends and family, feeling lonely and feeling optimistic about the future during the COVID-19 pandemic by different population groups

Changes in quality of life related factors were assessed with the question “Have the coronavirus pandemic or the subsequent restrictive measures affected your everyday life?” Majority of respondents reported having less interaction with friends and family due to the COVID-19 pandemic (Table 3). Negative changes were more often reported among women than men, those with higher education compared to those with lower education, those living with someone versus living alone, those having a good financial situation compared to those who suffered from economic deprivation, and those who used Internet to run errands versus those who did not (Fig. 1; *p* < 0.001 for all except for economic deprivation, for which *p* = 0.03, obtained from chi-square test). Almost half of the respondents reported also having felt lonelier during the pandemic (Table 3). Feelings of loneliness were reported more often among women, those over 85 years old, those who lived alone, those encountering economic deprivation, not using Internet to run errands and those manifesting severe IADL difficulties (Fig. 1; *p* < 0.001 for all, except for Internet use, for which *p* = 0.007, obtained from chi-square test). For feeling optimistic about the future, not as many negative changes were reported. Instead, about half of the respondents perceived that COVID-19 pandemic did not affect their feelings about the future and about a fifth reported having increased optimism (Table 3). However, negative changes were more often reported among women, those encountering economic deprivation, those with higher education, living alone, and using Internet to run errands (*p* < 0.001 for all except for living alone, for which *p* = 0.002, obtained from chi-square test).

**Table 3:** Self-evaluated changes in quality of life related factors during the COVID-19 pandemic among 75-year-old or older FinSote 2020 survey participants (response rate 92–95%, *N* = 6916–7127)

	Negative (%)	No change (%)	Positive (%)	N/A (*N*)
Keeping in touch with friends and family	63	27	10	436
Feeling lonely	42	54	4	1222
Feeling optimistic about the future	32	47	22	751
Sleeping difficulty/nightmares	20	77	2	1458
Daily exercise	28	59	13	600
Alcohol use	8	89	3	3791

Persons choosing the response option “N/A” (not applicable) were excluded from the analysis, with *N* representing the initial number of persons in the raw data

### Self-evaluated changes in sleeping difficulties, daily exercise, and alcohol use

**Fig. 2:**
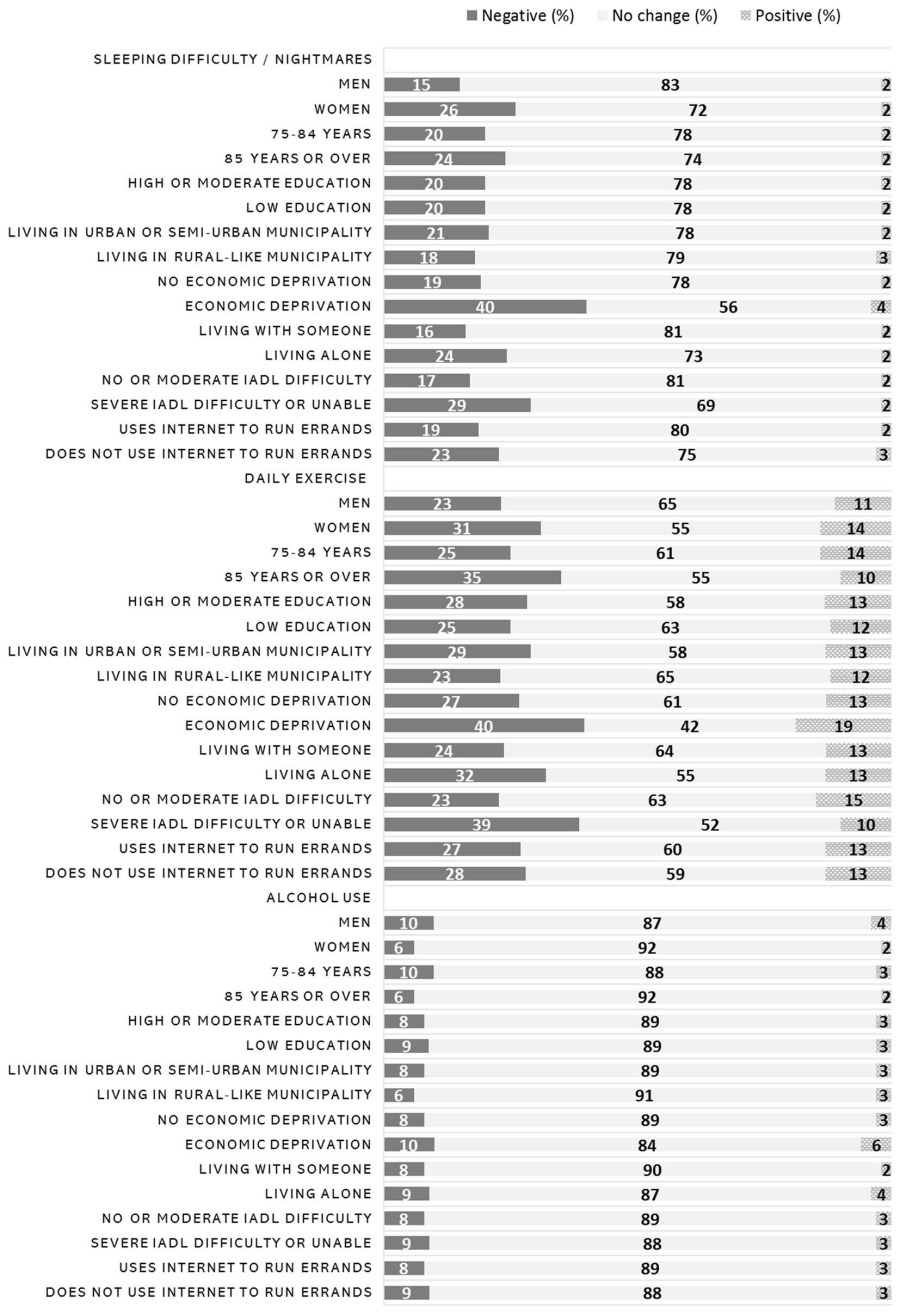
Older people's self-evaluated changes in sleeping difficulties, daily exercise, and alcohol use during the COVID-19 pandemic by different population groups

Although most respondents felt that the pandemic had no effect on their sleeping, one fifth still reported that sleeping difficulties and nightmares increased due to the pandemic (Table 3). Negative changes were more commonly reported by women, those encountering economic deprivation, those over 85 years old, those living alone, those with severe IADL difficulties and those not using Internet to run errands (Fig. 2; *p* < 0.001 for all, except for age and Internet use, for which *p* = 0.006 and *p* = 0.002, respectively, obtained from chi-square test). For daily exercise, both increases and decreases were observed: while slightly more than one fourth had decreased the amount of daily exercise, more than one in a ten had increased it (Table 3). Negative changes were more commonly reported by women, persons over 85 years old, those living alone, those manifesting severe IADL difficulty, those encountering economic deprivation, and those having higher education (Fig. 2; *p* < 0.001 for all except for education, for which *p* = 0.01, obtained from chi-square test). Also, those living in an urban or semi-urban area more often reported on decreased exercise levels due to the pandemic than those living in a rural-like area (*p* < 0.001, obtained from chi-square test). For alcohol use, nearly all participants reported that the pandemic had no effect on it (Table 3). The few negative changes were more commonly observed for men than women (Fig. 2; *p* < 0.001, obtained from chi-square test).

## Discussion

Our first aim was to assess whether Finnish older people’s QoL changed in Finland between the pre-pandemic year 2018 and 2020 and whether the change was different in various population groups by using data from nationally representative surveys from 2018 and 2020. We found a positive change in QoL index when data from 2018 and 2020 were compared and this was observed in nearly all population groups. Secondly, we were interested in changes in respondents’ self-rated quality of life related factors due to the pandemic in 2020. Many negative changes were reported, mainly in interaction with friends and relatives, feeling lonely, and having sleeping difficulties. These negative changes were more pronounced among women, those over 85 years of age, those encountering economic deprivation, manifesting severe IADL difficulty and those living alone, which implies that various socio-demographic features shape the effects of a global pandemic and its control measures on wellbeing. However, it was also found that some older people became more optimistic about the future and increased their daily exercise levels amid the pandemic.

Older people’s QoL is known to react to changes in health and social relationships in particular [3], which both were threatened during the COVID-19 pandemic. However, we observed no notable change in the QoL index scores when comparing data from pre-pandemic year 2018 to data from 2020. Instead, the scores remained consistent with the national reference values (3.8 among 75 years or older) [25] and even improved slightly over the 2 years, although the effect size was small. This finding is in line with previous papers reporting on the early-phase pandemic [6, 17, 26], and implies of older people’s high level of resilience, i.e., ability to adapt to hardship and cope with challenging life events, such as the COVID-19 control measures [27]. Older adults are often able to utilize proactive coping strategies, i.e., manage challenges before they become more stressful [28, 29], for example by focusing on positive thinking [30] and compensating restricted activities with something more feasible, e.g., physical activities outdoors [19, 26]. Furthermore, older people possess various life histories and experiences, e.g., from wartimes, other epidemics or personal life challenges, from which to draw upon [31], and they may also lean on self-enhancing comparisons, e.g., reflect their own situation to those in poorer conditions [29]. These strategies may help them to retain not only their sense of control and self-image [29], but also their level of satisfaction with their life even amid an adversity. Finally, it must be noted that when the present data were collected in 2020, the infection control measures were relaxed, and in 2021, at the end of the data collection period, the vaccinations started in the present age group. This may partially explain the findings.

Although the QoL index slightly improved from 2018 to 2020, many negative changes were self-reported in QoL-related factors. As reported in the early-phase pandemic (e.g., [6, 13–16]), also in the present study utilizing data from the early-phase pandemic, the most remarkable changes were centered on decreases in social activity and increases in loneliness and sleeping difficulties. Furthermore, we found that some population groups were more susceptible to the pandemic’s negative effects than others. Women, those over 85 years of age, those living alone, those suffering from economic deprivation and those manifesting severe IADL difficulties were identified as particularly vulnerable groups for diminished wellbeing in the COVID-19 era. While age, female sex and living alone have been identified as risk factors for compromised wellbeing also in previous studies (e.g., [15, 29, 32]), this was among the first studies to report on the difficulties faced by people with less economical resources. People encountering economic distress have fewer resources to cope with and adapt to unexpected life events and are at a greater risk for adverse effects related to health and lifestyle [33]. This was strongly manifested in our study. Finland is a Nordic welfare state with high coverage public health care and social welfare services (including income support) for all. Still, those older people who have encountered economic difficulties seemed not to be sufficiently protected against unexpected changes amid the pandemic. People with limited physical abilities have fewer possibilities to compensate for restricted activities with something more feasible, e.g., enjoying the outdoors [34, 35], as they often have difficulties to leave their homes and are dependent on external assistance and support to be physically active. During the COVID-19, this kind of assistance and services were contracted, or ceased altogether, because of social distancing and health security measures. Further, people with advanced functional limitations typically suffer from other functional and health deficits, such as cognitive decline [36], which may further reduce their opportunities for desired behavior amid the restrictions, but also make them a high-risk group for severe COVID-19 infections.

The use of Internet, in turn, was assumed to be a protective factor against the adverse effects of the pandemic as some of the activities and services that were run down during COVID-19 could be reached through the web. However, in contrast to our expectations, those older persons who reported using Internet reported also more negative changes, especially in their social activity, compared to those who did not use Internet. From this finding, we may deduce that the use of Internet in old age correlates with more active social life and perhaps better resources overall. While the pandemic seemed to have a particularly negative effect on the most vulnerable population groups, this finding implies that it also affected the lives of people who lead an active and social lifestyle prior to the pandemic.

Not all older people reacted negatively to the pandemic and its related control measures. For example, 22% of the participants in the present study reported that their optimism about the future increased and 13% reported that they had increased their daily levels of physical activity. As has been noted earlier [19, 35], it is likely that physical activity, particularly performed close to home, was among the most favorable ways to compensate for different restricted activities amid the COVID-19 pandemic. We also found that people living in rural municipalities reported less negative changes in their daily exercise levels compared to their urban counterparts. This may be because in rural areas outdoor space is more accessible for exercise and leisure activities and imply that large open spaces in rural environments make it easier to comply with the social distancing recommendations. Compared with the preliminary findings reported in the Finnish general population (terveytemme.fi/finsote/korona2020/), it seems that older people in the current study reported similar increases in daily exercise during the pandemic (15% vs. 13%, respectively). In contrast, older people seemed to report less decreases in optimism about the future than the general Finnish population (32% vs. 36%, respectively). A similar finding regarding hopefulness was found earlier as well [15]. Older people’s greater optimism may be explained by their vast life experience and resilience. It could also be expected that the everyday lives of older people, who are already retired and maybe adapted to being at home a lot, were not as affected as those of people at working age or with little children. Putting the findings to a broader context, Finland, being a Nordic welfare state, is characterised by efficient public health care services and social welfare to ensure peoples wellbeing and financial livelihood [12]. Finally, why some older people reacted more positively to the pandemic than others may be explained by certain personality traits, e.g., higher extraversion, and psychosocial resources, e.g., better stress-coping ability, as suggested before [37–39].

### Strengths and limitations

The present study was a large population-based, nationally representative survey, which included data on many socioeconomic and social background factors and allowed analyzing different population groups separately. Overall, there is little information from nationally representative samples on how the COVID-19 pandemic has affected to the quality of older people’s life, taken different population groups into account. The oldest old (over 85 years; 28% in the weighted sample) were well-represented within the study compared to many earlier studies on older adults. All analyses were conducted using weighted coefficients, so the data were nationally representative, and the findings generalizable to the whole Finnish older population, and likely to other western countries with similar COVID-19 situation. While the existing COVID-19 literature on older people’s QoL has focused on reporting the situation amid the first wave of the pandemic (e.g., [6, 17, 26]), the present study could utilize data collected at a time when the control measures had been in place for over a half a year to a year.

The major limitation in our study, however, is that it is a repeated cross-sectional survey and not a longitudinal investigation. Hence, although we could compare QoL-scores from year 2018 and 2020 and speculate the plausible changes, it is impossible to draw conclusions on causality and on the primary factors explaining the changes. Secondly, the EUROHIS-QoL 8-item index used here is composed of eight items representing psychological, physical, social, and environmental aspects of live. The concept of quality of life is multidimensional in nature, and thus the unidimensional nature of the index may not reveal a change in a specific area of life. The EUROHIS-QoL 8-item index is, however, recommended for use in public health research and has a good validity [40, 41]. In addition, as the data were collected with postal and online questionnaires, it is possible that self-reporting bias, such as social desirability bias and recall bias [42], have affected the results to some extent. On the other hand, having these two data collection methods has likely diminished the possibility that older people who use Internet, and perhaps are in better function, are over-represented. Concerning statistical analysis, it should be noted that we did not make corrections for the *p* values although many tests were conducted. However, given the large sample size and *p* value being < 0.001 in majority of the tests, this is not likely to change the results. Finally, although the study was population-based, it must be noted that older people with the poorest health and function, e.g., with an advanced cognitive impairment, were not well presented in the current study and hence, the findings of this study may not be generalizable to them.

## Conclusions

We found that quality of life (QoL) of Finnish older persons, assessed with the EUROHIS-QoL-8 index, improved slightly when pre-pandemic year 2018 and 2020 were compared. However, many negative changes were observed in QoL-related factors, such as keeping in touch with friends and relatives, feelings of loneliness, and sleeping. The negative changes due to the pandemic seemed to be more pronounced among women, the oldest old, those reporting economic deprivation, those living alone and those having severe IADL limitations, making these population groups especially vulnerable for diminished wellbeing in the COVID-19 era and potential target groups for future interventions. As this study reports findings from a repeated cross-sectional survey, the effects of the prolonged COVID-19 pandemic on QoL of older people should be confirmed in future with longitudinal investigations. In addition, future studies should examine whether the reported negative changes in different QoL-related factors are temporary, and behaviors returned to their initial levels as the pandemic has been solved.

## Declarations

### Data availability

The datasets generated and analyzed during the current study are not publicly available due to participant privacy requirements and the European general data protection regulation (GDPR). Pseudonymized data are available from the corresponding author on reasonable request.
